# Aspirin is associated with improved 30-day mortality in patients with sepsis-associated liver injury: a retrospective cohort study based on MIMIC IV database

**DOI:** 10.3389/fphar.2025.1514392

**Published:** 2025-03-04

**Authors:** Jianbao Wang, Xuemei Hu, Susu Cao, Yiwen Zhao, Mengting Chen, Tianfeng Hua, Min Yang

**Affiliations:** ^1^ The Second Department of Critical Care Medicine, The Second Affiliated Hospital of Anhui Medical University, Hefei, Anhui, China; ^2^ Laboratory of Cardiopulmonary Resuscitation and Critical Care, The Second Affiliated Hospital of Anhui Medical University, Hefei, Anhui, China; ^3^ Department of Pediatrics, The Second Affiliated Hospital of Anhui Medical University, Hefei, Anhui, China

**Keywords:** sepsis, sepsis-associated liver injury, aspirin, platelet, mortality

## Abstract

**Background:**

Sepsis-associated liver injury (SALI) is a common complication in sepsis patients, significantly affecting their prognosis. Previous studies have shown that aspirin can improve the prognosis of septic patients. However, there is currently a lack of clinical evidence supporting the use of aspirin in the treatment of SALI. Therefore, we conducted this study to explore the association between the use of aspirin and the prognosis of patients with SALI.

**Methods:**

The patients in this study were obtained from the Medical Information Mart for Intensive Care IV (MIMIC-IV) database, version 3.0. The primary outcome was 30-day all-cause mortality. Baseline characteristics between the aspirin and non-aspirin groups were balanced using propensity score matching (PSM). The Kaplan-Meier survival curve and Cox regression analysis were used to investigate the association between aspirin use and the prognosis of patients with SALI.

**Results:**

Of 657 SALI patients in this study, 447 (68%) patients had not used aspirin during hospitalization, whereas 210 (32%) had. After PSM, the 30-day mortality was 33.1% in the non-aspirin group and 21% in the aspirin group, indicating a significantly reduced mortality risk in the aspirin group (HR, 0.57; 95% CI, 0.37–0.90; *P* = 0.016). Similarly, the results of the multivariable Cox regression analysis and inverse probability weighting (IPW) analysis showed that, compared to the non-aspirin group, the aspirin group had a significantly lower 30-day mortality risk (Multivariable Cox regression analysis: HR, 0.69; 95% CI, 0.48–0.99; *P* = 0.047; IPW: HR, 0.62; 95% CI, 0.43–0.89; *P* = 0.010).

**Conclusion:**

Aspirin can reduce 30-day mortality in SALI patients, regardless of the dose or timing of administration. However, careful assessment based on individual differences is essential to ensure the safety and effectiveness of aspirin use.

## 1 Introduction

Sepsis is a common critical illness in intensive care units (ICU), characterized by a dysregulated host response to infection (Singer et al., 2016; Giamarellos-Bourboulis et al., 2024). A study indicated that the global mortality of sepsis patients is close to 20%, making it one of the major disease burdens on global health (Rudd et al., 2020). The main pathophysiological mechanism of sepsis involves the interaction between inflammation and coagulation (Ghasemzadeh and Hosseini, 2013). During sepsis, the coagulation system is activated at the site of infection (Engelmann and Massberg, 2013). Firstly, thrombin and thromboxane A2 (TXA2), two potent platelet activators, promote platelet activation by binding to protease-activated receptors (PAR) and TXA2 receptors, respectively (Engelmann and Massberg, 2013; Wang et al., 2018). Secondly, in the process of endothelial cell injury and apoptosis induced by sepsis, von Willebrand factor (vWF), expressed during these events, further enhances platelet activation and aggregation through glycoprotein receptors (GPVI and GPIbα--IX-V) on the platelet membrane (Engelmann and Massberg, 2013; Wang et al., 2018). This imbalance in the “platelets-inflammatory cell-endothelial cell” interaction plays a crucial role in the development of disseminated intravascular coagulation (DIC) and multiple organ dysfunction syndrome (MODS) (Wang et al., 2018). Therefore, by inhibiting platelet activation and reducing the interaction between “platelets-inflammatory cells-endothelial cells”, it may help block the “inflammation-coagulation cascade”, thereby reducing the consumption of platelets and coagulation factors. Additionally, platelets play a vital role in the activation, expansion, and regulation of inflammation (Eisen, 2012; Jung et al., 2012). Platelets also directly kill pathogens by releasing antimicrobial peptides, and enhance host antibacterial capacity by forming platelet-neutrophil extracellular traps (NETs) (Nicolai et al., 2024). Excessive platelet activation may lead to microthrombosis and instead impair tissue perfusion (Yang et al., 2023; Nicolai et al., 2024). Thus, aspirin may improve the immune balance at the site of infection by moderately inhibiting platelet function. Aspirin is the most widely used antiplatelet drug, and it also has anti-inflammatory and immunomodulatory effects (Montinari et al., 2019; Menter and Bresalier, 2023). In septic patients, the use of aspirin can improve their prognosis (Hsu et al., 2022; Chen et al., 2023; Lu et al., 2023; Dong et al., 2024). In addition to exerting antiplatelet and anti-inflammatory effects by inhibiting cyclooxygenase (COX-1 and COX-2), aspirin can also regulate the inflammatory response by inhibiting the NF-κb inflammatory pathway (Ornelas et al., 2017). Moreover, acetylsalicylic acid, a metabolite of aspirin, can inhibit High Mobility Group Box 1 (HMGB1), thereby suppressing the inflammatory response (Venereau et al., 2016). Therefore, aspirin has significant potential to improve the prognosis of infectious diseases by modulating inflammatory responses and coagulation functions.

The liver, a crucial lymphoid organ, is pivotal in regulating immune defense by clearing bacteria or toxins, producing acute-phase proteins or cytokines, and regulating inflammatory metabolism (Nesseler et al., 2012; Yan et al., 2014; Strnad et al., 2017). However, the immune response is a double-edged sword, as an excessive systemic inflammatory response may lead to liver injury (Nesseler et al., 2012; Yan et al., 2014; Strnad et al., 2017). The liver is also a target of sepsis-induced injury, including hypoxic hepatitis caused by shock or hypoxia, cholestasis resulting from dysregulated bile metabolism, hepatocellular damage from drug toxicity, and secondary sclerosing cholangitis (Strnad et al., 2017). Liver dysfunction caused by sepsis is an independent risk factor for multi-organ dysfunction and sepsis-induced mortality (Yan et al., 2014). Sepsis-associated liver injury (SALI) is characterized by INR >1.5, highlighting the critical role of coagulopathy in its pathology (Dellinger et al., 2013; Xie et al., 2022; Cui et al., 2023; Yi et al., 2024). Aspirin’s anti-inflammatory and antiplatelet properties can help reduce inflammation responses and the risk of DIC(Wang et al., 2018; Al-Husinat et al., 2023), which may play a positive role in modulating the inflammatory responses and coagulation function in SALI. Jiang et al. also found that aspirin can improve the prognosis of patients with SALI by regulating the homeodomain-interacting protein kinase 2 (HIPK2) (Jiang et al., 2018). Therefore, investigating the relationship between aspirin use and the prognosis of SALI patients is crucial, as it helps to elucidate its potential benefits in mitigating inflammatory responses and coagulation abnormalities. To this end, we conducted this study to explore the association between aspirin use and the prognosis of SALI patients.

## 2 Materials and methods

### 2.1 Data source

The study’s data was exclusively sourced from the Medical Information Mart for Intensive Care IV database, version 3.0 (MIMIC-IV, v3.0) (Johnson et al., 2023; Johnson et al., 2024). The database provides clinical data on all patients admitted to the ICU at the Beth Israel Deaconess Medical Center from 2008 to 2021. Authors who retrieve data from databases have completed the Protecting Human Research Participants course on the National Institutes of Health website and obtained certification (NO.10756634) before accessing the data. The SQL code for extracting the data is from https://github.com/MIT-LCP/mimic-code/.

### 2.2 Study patients

We initially included patients who met the criteria for sepsis 3.0 on the first day of ICU admission (Singer et al., 2016). According to the Surviving Sepsis Campaign Guidelines and previous studies, the diagnostic criteria for SALI in this study were INR >1.5 and total bilirubin >2 mg/dL (34.2 μmol/L) in septic patients within the first 24 h of ICU admission (Dellinger et al., 2013; Xie et al., 2022; Cui et al., 2023; Yi et al., 2024). The Exclusion criteria were: (1) Hospital and ICU readmission; (2) Age <18 years old; (3) ICU length of stay <24 h; (4) All liver diseases.

### 2.3 Variables

The following demographic variables were extracted from the first ICU admission: age, gender, and race. White blood cell (WBC), hemoglobin, platelets, lymphocyte count, neutrophils count, monocyte count, international normalized ratio (INR), alanine aminotransferase (ALT), aspartate aminotransferase (AST), alkaline phosphatase (ALP), lactate dehydrogenase (LDH), fibrinogen, albumin, lactate, partial pressure of arterial oxygen (PaO_2_), partial pressure of arterial carbon dioxide (PaCO_2_), heart rate, respiratory rate, temperature, mean blood pressure (MBP), and oxygen saturation (SPO_2_) measured on the first day of ICU admission were extracted. In addition, the Patients’ comorbidities, Charlson comorbidity index (CCI), critical treatments on the first day of ICU admission (RRT, mechanical ventilation, and vasopressor), and severity scores including sequential organ failure assessment score (SOFA) and simplified acute physiology score II (SAPS II) were extracted. Indicators representing multiple records by calculating the worst values based on the direction of abnormality (Supplementary Table S1).

### 2.4 Study outcomes

The primary outcome was 30-day all-cause mortality. Secondary outcomes included 90-day all-cause mortality, in-hospital mortality, ICU mortality, duration of hospital stay, and duration of ICU stay.

### 2.5 Missing values and outliers of variables

There are many missing values and outliers of the variables (Supplementary Figure S1A; Supplementary Figure S6). We excluded variables with more than 25% missing values and imputed others by multiple imputations or the missForest method. The distributions of variables were compared between the imputed cohorts and the original cohort using joy plots, and the imputed cohort most similar to the original cohort was selected for data analysis (Cart cohort) (Supplementary Figure S1B). For some outliers in the data, we perform Winsorize transformation and replace them with the boundary values—[Quantile 1–3×IQR](lower)/[Quantile 3 + 3×IQR](upper).

### 2.6 Propensity score matching

Patients with SALI were matched 1:1 using the nearest neighbor method, with a caliper width of 0.05 on the propensity score (PS) scale. The balance of confounding variables was evaluated using standardized differences, with significant imbalances defined as values exceeding 10% (Supplementary Figures S1D, E).

### 2.7 Statistical analysis

Count data were expressed as frequencies, and P-values for group comparisons were calculated using the chi-square test. Depending on the sample sizes, either Pearson’s chi-square test or Fisher’s exact test was used. The Shapiro-Wilk test was performed to assess the normality of continuous data. Data that followed a normal distribution were reported as mean ± standard deviation (SD), and comparisons between groups were conducted using the independent samples *t-test*. For data that did not meet the normality assumption, values were presented as median (interquartile range, IQR), with the Mann-Whitney U test employed for intergroup comparisons. Due to the multicollinearity between variables may reduce the accuracy of multivariate regression results, when two variables are highly correlated (Spearman coefficient >0.7), one variable with a lower correlation with the outcome is removed (Supplementary Figure S1B). We used multivariate COX regression analysis to examine the association between aspirin and mortality. Results are shown as Hazard Ratios (HR) and 95% confidence intervals (CI). The survival analysis was performed using the Kaplan-Meier survival curve. We also employed restricted cubic spline (RCS) regression models to examine the association between INR or total bilirubin levels and 30-day mortality. A restricted cubic spline model with three–seven nodes was fitted, and the model with the lowest Akaike Information Criterion (AIC) was selected to determine the optimal number of nodes. Subgroup analyses were performed as stratified by age, gender, race, INR, total bilirubin, baseline scores (SOFA score, SAPS II score, CCI score), comorbidities (hypertension, CAD, cerebrovascular disease, diabetes, and AKI), and critical treatments on the first day of ICU admission (RRT, mechanical ventilation, and vasopressor). A *P* value <0.05 for two sides is considered statistical significance. Structure Query Language (SQL) and Navicat Premium (version 17.0) were used to extract raw data. Statistical analysis was performed using R software (version 4.4.0).

## 3 Results

### 3.1 Baseline characteristics of patients with SALI

**FIGURE 1:**
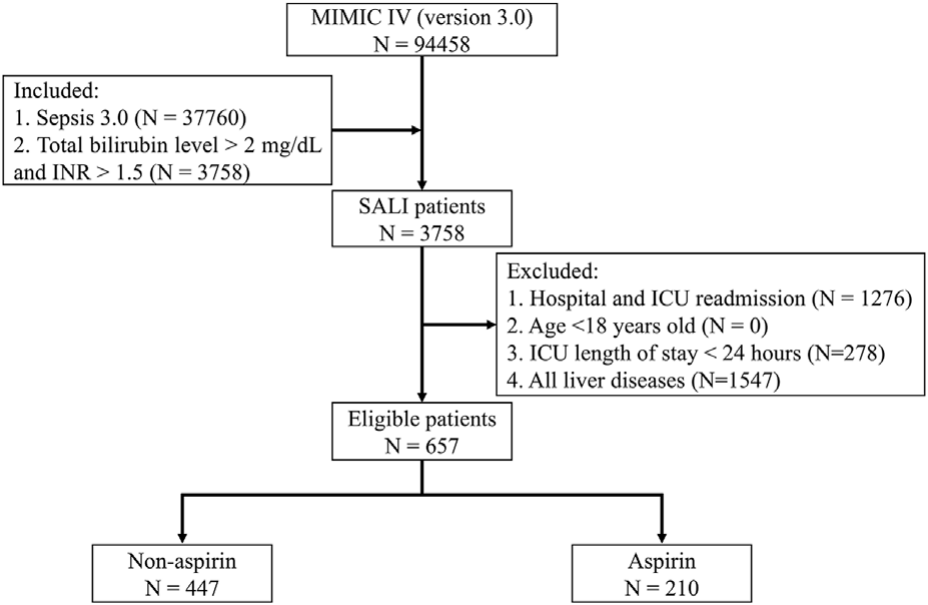
Flow Chart. MIMIC IV: Medical Information Mart for Intensive Care IV; ICU: Intensive Care Unit; SALI: Sepsis-associated liver injury; INR: International Normalized Ratio.

Of 657 SALI patients in this study, 447 (68%) patients had not used aspirin during hospitalization, whereas 210 (32%) had (Figure 1). SALI patients in the non-aspirin had a median age of 69.1 years, 55.5% were male, 60.2% were white, the median INR was 2.1, and the median total bilirubin was 3.7 mg/dL. In the aspirin group, the median age was 73.1 years, 69.1% were male, 66.2% were white, the median INR was 1.9, and the median total bilirubin was 3.4 mg/dL. There were significant age differences in age (*P* = 0.002), gender (*P* < 0.001), and total bilirubin (*P* = 0.009) between the two groups. For comorbidities, the aspirin group had a significantly higher proportion of each comorbidity compared to the non-aspirin group, while there was no significant difference in SOFA score, SAPS II score, and treatment measures on the first day between the two groups. After PSM, the baseline characteristics of the two groups were generally balanced (Table 1).

**TABLE 1:** Characteristics of patients with SALI.

Variables	Before PSM			After PSM		
	Non-aspirin	Aspirin	P value	Non-aspirin	Aspirin	P value
Patients number	447	210		148	148	
Demographics						
Age	69.2 (56.8, 81.4)	73.1 (64.1, 81.5)	0.002	75.1 (58.7, 83.6)	72.2 (62.6, 80.0)	0.389
Gender, male	248 (55.5)	145 (69.1)	<0.001	91 (61.5)	94 (63.5)	0.719
Race			0.127			0.870
Other/Unknown	130 (29.1)	58 (27.6)		47 (31.76)	43 (29.05)	
Black	48 (10.7)	13 (6.2)		9 (6.08)	10 (6.76)	
White	269 (60.2)	139 (66.2)		92 (62.16)	95 (64.19)	
Vital/Laboratory variables						
Temperature	37.3 (37.0, 38.1)	37.3 (37.0, 38.0)	0.962	37.3 (37.0, 38.0)	37.4 (37.0, 38.1)	0.362
Heart rate, bmp	117.0 (100.0, 132.0)	108.0 (94.3, 124.0)	<0.001	113.4 ± 21.9	112.3 ± 21.5	0.655
Respiratory Rate	30.0 (26.0, 35.0)	28.0 (25.0, 34.0)	0.025	29.8 (26.0, 34.0)	28.0 (25.0, 34.3)	0.607
MBP, mmHg	55.0 (47.0, 62.0)	56.0 (47.3, 62.0)	0.781	55.0 (48.8, 61.3)	56.0 (47.8, 62.0)	0.729
SpO_2_	92.0 (88.0, 94.0)	92.0 (89.3, 94.0)	0.237	92.0 (89.0, 94.0)	92.0 (89.0, 94.0)	0.573
WBC	16.7 (10.4, 25.4)	16.8 (10.7, 22.8)	0.876	15.5 (10.3, 24.1)	16.8 (10.0, 23.0)	0.590
Platelets	115.0 (63.0, 176.0)	133.0 (83.3, 191.5)	0.003	135.5 (82.8, 192.8)	131.5 (81.0, 192.5)	0.782
Hemoglobin	9.5 (7.7, 11.1)	9.4 (7.7, 11.4)	0.601	9.7 (8.0, 11.4)	9.2 (7.7, 11.3)	0.322
Lymphocytes count	0.8 (0.4, 1.3)	0.8 (0.5, 1.4)	0.161	0.8 (0.5, 1.1)	0.9 (0.6, 1.3)	0.123
Monocytes count	0.6 (0.3, 1.1)	0.8 (0.4, 1.2)	0.003	0.6 (0.3, 1.1)	0.8 (0.4, 1.3)	0.046
Neutrophils count	11.9 (7.1, 18.9)	11.6 (7.7, 18.0)	0.691	11.7 (7.3, 18.5)	12.4 (7.5, 17.8)	0.600
INR	2.1 (1.8, 2.8)	1.9 (1.7, 2.7)	0.121	2.1 (1.8, 2.6)	1.9 (1.7, 2.8)	0.399
Total bilirubin	3.7 (2.6, 6.1)	3.4 (2.5, 4.7)	0.009	3.4 (2.6, 4.7)	3.5 (2.7, 5.0)	0.756
Albumin	2.71 ± 0.63	2.88 ± 0.56	<0.001	2.88 ± 0.62	2.84 ± 0.54	0.608
ALT	119.0 (41.0, 367.0)	118.5 (37.3, 289.8)	0.446	115.5 (40.0, 390.0)	121.5 (33.5, 296.8)	0.639
ALP	168.0 (91.5, 295.5)	126.5 (74.3, 250.8)	0.002	165.0 (92.5, 238.0)	141.0 (76.8, 261.8)	0.310
AST	172.0 (66.5, 577.0)	175.5 (78.5, 421.3)	0.829	181.0 (57.0, 610.8)	176.0 (80.0, 472.3)	0.917
CCI score	5.0 (3.0, 8.0)	6.0 (4.0, 8.0)	<0.001	6.0 (3.0, 8.0)	6.0 (4.0, 8.0)	0.601
SOFA score	9.0 (6.0, 12.5)	9.0 (7.0, 12.0)	0.532	9.0 (5.8, 12.0)	9.0 (7.0, 11.3)	0.690
SAPS II score	49.0 (39.0, 63.0)	47.5 (37.3, 58.8)	0.151	47.0 (36.8, 57.0)	47.0 (37.8, 58.0)	0.749
Comorbidity						
Hypertension	89 (20.0)	65 (31.0)	0.002	38 (25.7)	45 (30.4)	0.365
CAD	50 (11.2)	104 (49.5)	<0.001	49 (33.1)	47 (31.8)	0.804
Cerebrovascular	27 (6.0)	32 (15.2)	<0.001	19 (12.8)	21 (14.2)	0.734
Diabetes	116 (26.0)	77 (36.7)	0.005	50 (33.8)	48 (32.4)	0.805
AKI	294 (65.8)	162 (77.1)	0.003	105 (71.0)	109 (73.7)	0.603
Critical treatments on the first day						
RRT	43 (9.62)	15 (7.14)	0.297	16 (10.8)	12 (8.1)	0.427
Vasopressor	228 (51.01)	110 (52.38)	0.742	72 (48.7)	75 (50.7)	0.727
Mechanical ventilation	199 (44.52)	105 (50.00)	0.189	60 (40.5)	76 (51.4)	0.062

SALI: Sepsis-associated liver injury; MBP: mean blood pressure; SpO_2_: oxygen saturation; WBC: white blood cell; SOFA: sequential organ failure assessment; SAPS II: Simplified Acute Physiology Score II; INR: international normalized ratio; ALT: alanine aminotransferase; ALP: alkaline phosphatase; AST: aspartate aminotransferase; CCI: charlson comorbidity index; CAD: coronary artery disease; AKI: acute kidney injury; RRT: renal replacement therapy; PSM: Propensity Score Matching. *P* value <0.05 is considered statistical significance.

### 3.2 Clinical outcomes of patients with SALI

Table 2 shows the clinical outcomes of patients in both groups. After PSM, the non-aspirin group had a median hospital stay of 8.1 days, ICU stay of 2.8 days, in-hospital mortality of 29.1%, ICU mortality of 25.0%, 30-day mortality of 33.1%, and 90-day mortality of 41.9%. The aspirin group had a median hospital stay of 12.0 days, ICU stay of 4.5 days, in-hospital mortality of 23.7%, ICU mortality of 14.2%, 30-day mortality of 21%, and 90-day mortality of 33.8%. Compared to the non-aspirin group, the aspirin group had significantly longer length of hospital (*P* < 0.001) and ICU stays (*P* < 0.001) but significantly lower ICU (*P* = 0.019) and 30-day (*P* = 0.018) mortality. Before PSM, the clinical outcomes of both groups also supported this result.

**TABLE 2:** Clinical outcomes of patients with SALI.

Variables	Before PSM			After PSM		
	Non-aspirin	Aspirin	P value	Non-aspirin	Aspirin	P value
Patients number	447	210		148	148	
Length of hospital stay	8.9 (4.5, 17.0)	11.0 (6.1, 19.0)	0.020	8.1 (4.0, 14.8)	12.0 (7.2, 19.1)	<0.001
Length of ICU stay	3.0 (1.8, 5.8)	4.0 (2.2, 7.2)	0.003	2.8 (1.8, 5.2)	4.5 (2.5, 7.7)	<0.001
In-hospital mortality	147 (32.9)	58 (27.6)	0.174	43 (29.1)	35 (23.7)	0.291
ICU mortality	129 (28.9)	40 (19.1)	0.007	37 (25.0)	21 (14.2)	0.019
30-day mortality	163 (36.5)	56 (26.7)	0.013	49 (33.1)	31 (21.0)	0.018
90-day mortality	199 (44.5)	79 (37.6)	0.095	62 (41.9)	50 (33.8)	0.150

SALI: Sepsis-associated liver injury; PSM: propensity score matching; ICU: Intensive Care Unit. *P* value <0.05 is considered statistical significance.

### 3.3 Survival analysis

**FIGURE 2:**
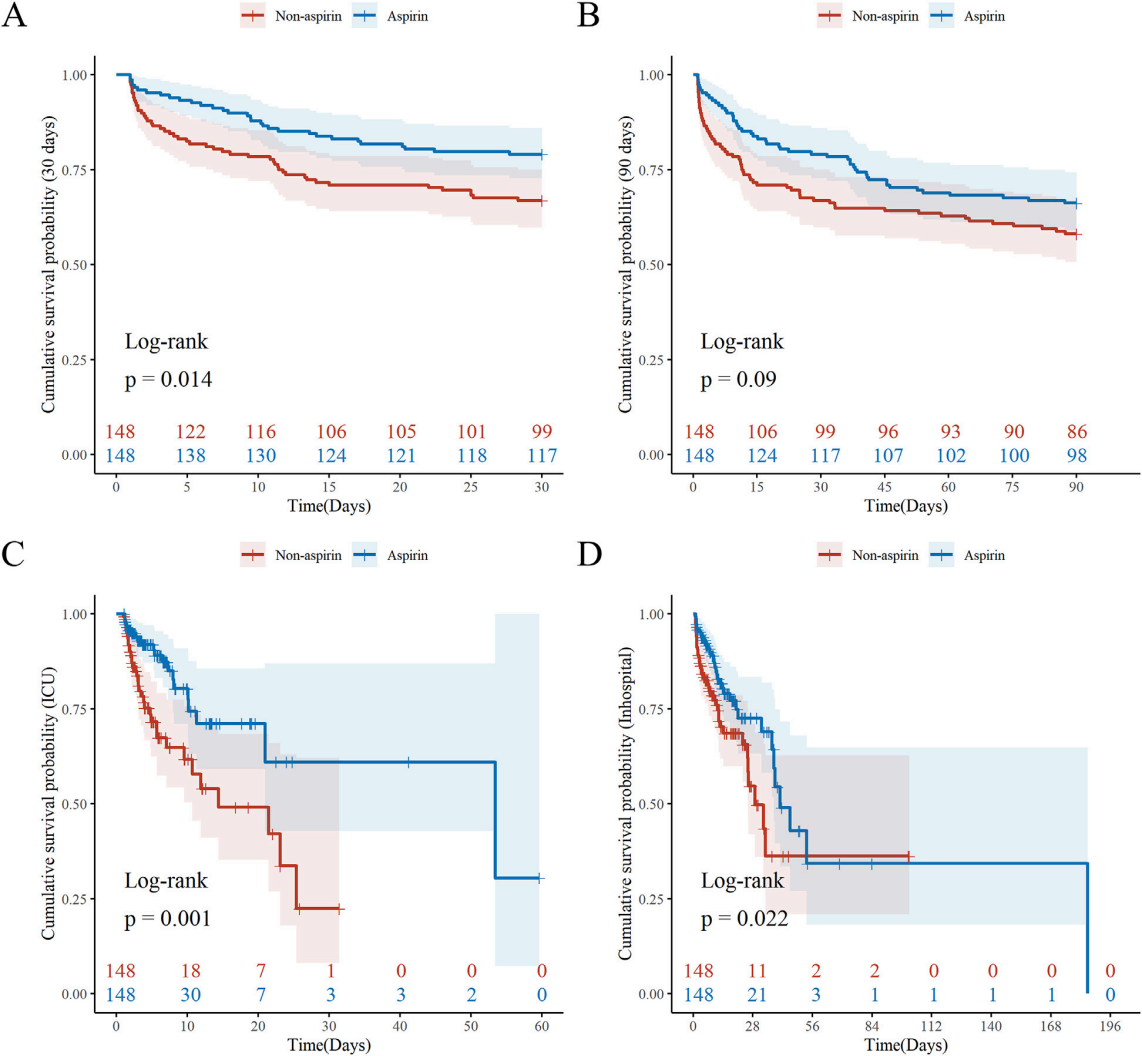
Kaplan-Meier Survival Analysis by Aspirin after PSM. Kaplan–Meier curves (log-rank test) are plotted for 30-day mortality **(A)**, 90-day mortality **(B)**, ICU mortality **(C)**, and in-hospital mortality **(D)**, grouped by aspirin use. The X-axis denotes the time (days) in ICU and the Y-axis denotes the cumulative survival probability. ICU: Intensive Care Unit; PSM: Propensity Score Matching. *P* value <0.05 is considered statistical significance.

**FIGURE 3:**
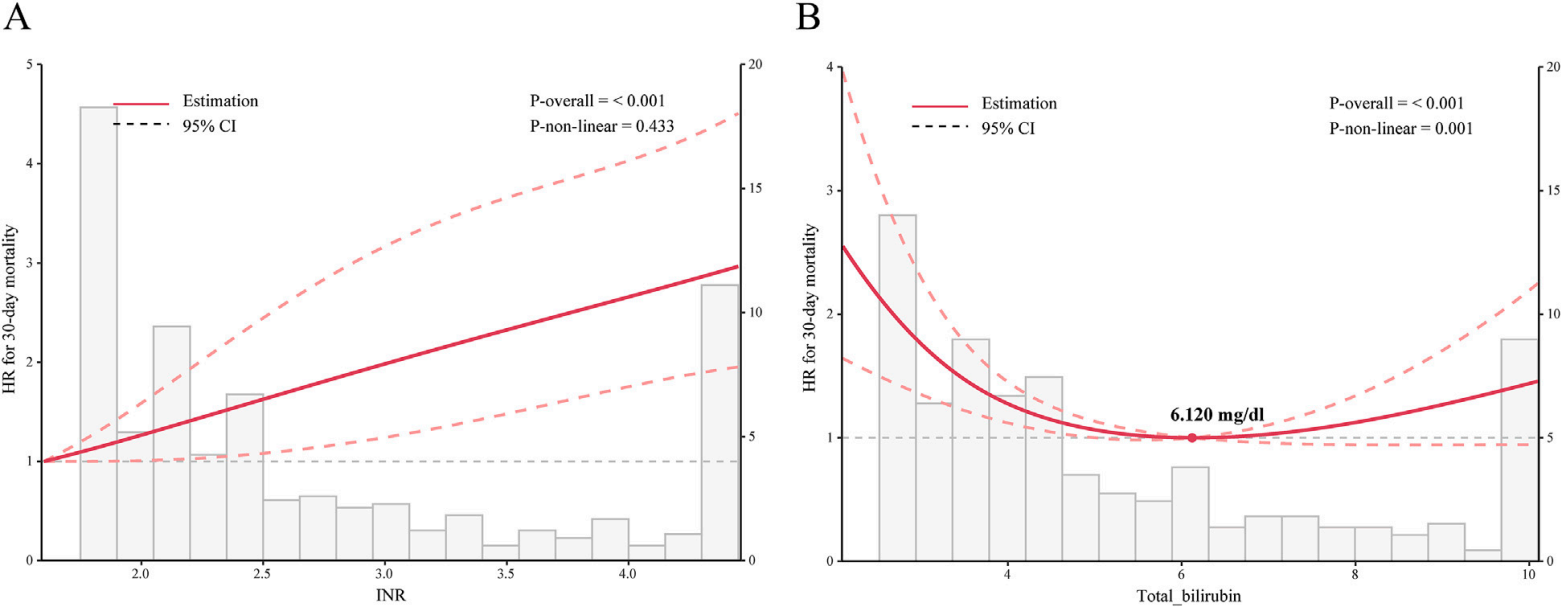
Relationship Between INR or Total Bilirubin and 30-day Mortality in Patients with SALI. Graphs show HRs between INR **(A)** or total bilirubin **(B)** and 30-day mortality, adjusted for age, gender, race, platelets, albumin, ALP, AST, CCI, SOFA score, SAPS II score, hypertension, CAD, cerebrovascular, diabetes, AKI, RRT, vasopressor, and mechanical ventilation. Data were fitted by a restricted cubic spline Cox proportional hazards regression model, and the model was conducted with 4 knots. Solid lines indicate HRs, and shadow shapes indicate 95% CIs. INR: International Normalized Ratio; HR: hazard ratio; CI: confidence interval. *P* value <0.05 is considered statistical significance.

Kaplan-Meier survival curves showed the cumulative survival probabilities over time for both groups. Before PSM, the aspirin group had significantly higher 30-day (*P* = 0.01), 90-day (*P* = 0.049), and ICU (*P* < 0.001) cumulative survival probabilities compared to the non-aspirin group. After PSM, the aspirin group had significantly higher 30-day (*P* = 0.014), ICU (*P* = 0.001), and in-hospital (*P* < 0.022) cumulative survival probabilities (Figure 2; Supplementary Figure S2). Both PSM and multivariate Cox regression analysis indicated that the use of aspirin was an independent protective factor for 30-day mortality in patients with SALI (PSM: HR, 0.57; 95% CI, 0.37–0.90; *P* = 0.016; Multivariate Cox regression analysis: HR, 0.69; 95% CI, 0.48–0.99; *P* = 0.047). Furthermore, through inverse probability weighting (IPW) analysis to enhance the robustness of the results, it was also found that patients in the aspirin group had a lower 30 - day mortality risk (HR, 0.62; 95% CI, 0.43–0.89; *P* = 0.010) (Table 3). In addition, baseline INR and total bilirubin levels were significantly correlated with mortality in patients with SALI. The RCS results showed a linear relationship between INR and 30-day mortality, while total bilirubin had a “U-shaped” nonlinear relationship (Figure 3).

**TABLE 3:** Association between aspirin treatment and mortality of patients with SALI.

Mortality	Model	HR (95% CI)	P value
30-day mortality	Unadjusted	0.67 (0.50 ∼ 0.91)	0.011
	PSM	0.57 (0.37 ∼ 0.90)	0.016
	Multivariate adjusted	0.69 (0.48 ∼ 0.99)	0.047
	IPW	0.62 (0.43 ∼ 0.89)	0.010
90-day mortality	Unadjusted	0.77 (0.59 ∼ 0.99)	0.049
	PSM	0.73 (0.50 ∼ 1.05)	0.091
	Multivariate adjusted	0.79 (0.58 ∼ 1.08)	0.141
	IPW	0.74 (0.54 ∼ 1.00)	0.051
ICU mortality	Unadjusted	0.53 (0.37 ∼ 0.76)	<0.001
	PSM	0.41 (0.24 ∼ 0.71)	0.001
	Multivariate adjusted	0.61 (0.39 ∼ 0.95)	0.030
	IPW	0.43 (0.28 ∼ 0.67)	<0.001
In-hospital mortality	Unadjusted	0.74 (0.55 ∼ 1.01)	0.058
	PSM	0.59 (0.37 ∼ 0.93)	0.024
	Multivariate adjusted	0.88 (0.61 ∼ 1.27)	0.491
	IPW	0.67 (0.47 ∼ 0.95)	0.024

Non-aspirin is reference. Multivariate adjusted: Adjusted for age, gender, race, platelets, albumin, INR, total bilirubin, ALP, AST, CCI, SOFA, score, SAPS II, score, hypertension; CAD, cerebrovascular, diabetes, AKI, RRT, vasopressor, and mechanical ventilation. SALI: Sepsis-associated liver injury; SOFA: sequential organ failure assessment; SAPS II: Simplified Acute Physiology Score II; INR: international normalized ratio; ALP: alkaline phosphatase; AST: aspartate aminotransferase; CCI: charlson comorbidity index; CAD: coronary artery disease; AKI: acute kidney injury; RRT: renal replacement therapy; PSM: propensity score matching; IPW: Inverse - Probability Weighting; ICU: intensive care unit; HR: hazard ratio; CI: Confidence Interval. *P* value <0.05 is considered statistical significance.

To explore the association between the dosage of aspirin and mortality in patients with SALI, patients using aspirin were divided into high-dose (55 patients) and low-dose (155 patients) groups based on whether the dosage exceeded 81 mg. Kaplan-Meier survival curves showed that there was no significant difference in the cumulative survival probability between the two groups (Supplementary Figure S3). To further investigate the association between the timing of aspirin use and mortality in patients with SALI, patients using aspirin were divided into the pre-ICU group (52 patients) and the post-ICU group (158 patients). The KM survival curves showed that patients who received aspirin post-ICU had a higher cumulative probability of survival (30-day, 90-day, ICU) (Supplementary Figure S4). After adjustment, the timing of aspirin administration was associated only with ICU mortality (Supplementary Table S2).

### 3.4 Absolute platelet changes and incidence of DIC

We have found that the change in platelets in the aspirin group was significantly lower than that in the non - aspirin group (8.82 vs. 12.36 × 10^3^/μL, P = 0.04) (Supplementary Table S3; Supplementary Figure S7). The incidence of DIC was lower in the aspirin group than in the non - aspirin group (4.3% vs. 8.3%), and the mortality in the aspirin group was also lower (Supplementary Figure S7).

### 3.5 Subgroup analysis

This study used a stratified Cox proportional hazards model for subgroup analysis, with the non-aspirin group as the common control. For continuous variables, all were divided based on the median, except for age, which was categorized into two subgroups at 65 years. The subgroup analysis showed that aspirin was more effective in SALI patients who were over 65 years old, white, INR ≤2.1, SOFA scores >9, SAPS II scores >47, without hypertension, without CAD, with cerebrovascular disease, without diabetes, with AKI, without RRT, and who were receiving vasopressors or mechanical ventilation after PSM (Supplementary Figure S5).

## 4 Discussion

In this study, we found that aspirin was significantly associated with 30-day mortality in patients with SALI, regardless of aspirin dose and timing. Previous studies reported that the mortality of SALI patients was approximately 29.3%–43.5% (Liu et al., 2022; Wen et al., 2024; Yi et al., 2024). Consistent with previous studies, our study found that the 30-day mortality of SALI patients was 33.3% (219/657), with 36.5% in the non-aspirin group and 26.7% in the aspirin group. The use of aspirin significantly improved the prognosis of SALI patients.

A population-based cohort study conducted by Hsu et al., with a median follow-up of 6.2 years involving 29,690 participants, found that aspirin use did not improve the long-term outcomes of sepsis patients (Hsu et al., 2018). Similar to the results of Hsu et al., another large randomized controlled trial conducted by Eisen et al. included 16,703 sepsis patients aged 70 years and older (Eisen et al., 2021). After a median follow-up of 4.6 years, the study results showed that, compared to the control group, patients receiving low-dose aspirin did not show lower mortality (HR, 1.08; 95%CI, 0.82–1.43; *P* = 0.57), but increased risk of gastrointestinal bleeding and hemorrhagic stroke. Therefore, Eisen et al. did not support the use of aspirin as a preventive measure for sepsis. However, Annane highlighted the following limitations in these two studies (Annane, 2021): First, both studies diagnosed sepsis using the Systemic Inflammatory Response Syndrome (SIRS) criteria, which lack precision and may have led to the exclusion of some sepsis patients and the inclusion of a considerable number of non-sepsis patients. Second, the follow-up duration might still have been too short to fully observe the long-term effects of aspirin on septic patients. Therefore, Annane believed that the potential impact of aspirin on the long-term outcomes of sepsis patients cannot be completely ruled out. Recent studies on aspirin and sepsis have suggested that the use of aspirin can reduce the 28-day, 60-day, 90-day, and 1-year mortality in sepsis patients with damage to various organs (Chen et al., 2023; Lu et al., 2023; Dong et al., 2024). However, our study only found that aspirin could reduce 30-day mortality and ICU mortality, indicating an effect on short-term outcomes in SALI patients. Trauer et al. conducted a meta-analysis of previous studies of aspirin intervention in sepsis and showed that aspirin reduced short-term mortality in septic patients (Trauer et al., 2017). Based on the above, there are significant differences in the effects of aspirin on outcomes in septic patients. Most previous studies have suggested that aspirin fails to improve long-term outcomes in these patients (Trauer et al., 2017; Hsu et al., 2018; Annane, 2021). However, some recent studies have presented an opposite view (Chen et al., 2023; Lu et al., 2023; Dong et al., 2024). Whether aspirin improves long-term outcomes in septic patients remains to be further explored. According to our findings, we believe that aspirin can improve short-term outcomes in septic patients, which is consistent with other findings. We believe that these differences may be related to the diagnostic methods of sepsis, the presence of concomitant organ failure, and the underlying comorbidities of the patients.

Different doses of aspirin have different pharmacological effects (Patrono and Baigent, 2019). Low-dose aspirin primarily inhibits COX-1 to exert an antiplatelet effect, while high-dose aspirin exerts both antiplatelet and anti-inflammatory effects by inhibiting COX-1, COX-2, and NF-κb inflammatory pathway (Ornelas et al., 2017). Although Chen et al. suggested that both the antiplatelet and anti-inflammatory effects of aspirin can benefit sepsis patients, with high-dose aspirin being more effective than low-dose aspirin (Chen et al., 2023). However, our study did not observe any differences in the effects between high-dose and low-dose aspirin, which is consistent with the findings of most studies (Lu et al., 2023; Dong et al., 2024). Actually, low-dose aspirin can exert anti-inflammatory effects by triggering the synthesis of lipoxins (Eisen, 2012; Otto et al., 2013; Toner et al., 2015). Previous studies have shown that long-term use of low-dose aspirin before hospital admission is associated with reduced mortality in patients with sepsis (Tsai et al., 2015; Hsu et al., 2022). This may be because aspirin improves the outcomes of septic patients by enhancing the inflammatory response (Kiers et al., 2016). In addition, the use of aspirin during hospitalization can also reduce the mortality of septic patients (Ouyang et al., 2019; Wang et al., 2023; Dong et al., 2024). The impact of aspirin on the prognosis of septic patients may be independent of the timing of administration. Interestingly, our study found that reduced 30-day mortality in patients with SALI was independent of the timing of aspirin administration, but ICU mortality was lower when administered in the post-ICU. Platelets play an important role in the activation, expansion, and regulation of inflammation, and can enhance host antimicrobial defense, however excessive platelet activation may lead to microthrombosis and instead impair tissue perfusion, so inhibition of platelets has important potential to improve the prognosis of infectious diseases (Eisen, 2012; Jung et al., 2012; Yang et al., 2023; Nicolai et al., 2024). We believe that long-term administration of aspirin before the onset of sepsis mainly reflects its preventive effect, which is primarily focused on enhancing the inflammatory response in sepsis patients. In contrast, the use of aspirin after the onset of sepsis demonstrates its therapeutic effect, mainly through its antiplatelet action.

The liver serves as the primary source of thrombopoietin (TPO) (Qian et al., 1998; Kaushansky, 2005). Liver injury frequently causes thrombocytopenia, which not only impairs coagulation but may also intensify inflammatory responses, thereby forming a “thrombocytopenia-inflammation” vicious cycle (Eisen, 2012; Jung et al., 2012).In sepsis, “platelets-inflammatory cell-endothelial cell” interaction can lead to thrombocytopenia (Engelmann and Massberg, 2013; Wang et al., 2018). In the presence of liver injury, this process further exacerbates “thrombocytopenia-inflammation” vicious cycle. In addition, during liver injury, high expression of COX-1 in liver sinusoidal endothelial cells increases thromboxane A2 production, resulting in endothelial dysfunction and microcirculatory disturbances in the liver (Lin et al., 2017; Gracia-Sancho et al., 2021). Aspirin may improve this situation by inhibiting local thromboxane A2 synthesis (Ornelas et al., 2017). Systemic inflammatory response is one of the important manifestations of sepsis (Giamarellos-Bourboulis et al., 2024). During liver injury, the liver’s ability to clear bacterial endotoxins is impaired, which may exacerbate the systemic inflammatory response (Nesseler et al., 2012; Yan et al., 2014; Strnad et al., 2017). Aspirin may exert a secondary anti-inflammatory effect by inhibiting HMGB1 (Venereau et al., 2016). Our study found that compared with the non - aspirin group, patients in the aspirin group had lower platelet changes and a lower incidence of DIC. Thus, our study proposes that the potential mechanisms by which aspirin improves outcomes in patients with SALI are as follows: 1. During infection, aspirin inhibits excessive platelet activation and reduces platelet consumption, thus slowing down the progression of the “thrombocytopenia - inflammation” vicious cycle; 2. Aspirin reduces the formation of microthrombi by inhibiting the synthesis of thromboxane A2 in the liver, improves the hepatic microcirculation, and slows down the further deterioration of liver injury; 3. In the setting of liver injury, aspirin exerts a secondary anti-inflammatory effect by inhibiting HMGB1, which alleviating the systemic inflammatory response.

Our subgroup analysis showed a better benefit of aspirin in certain specific SALI patients. However, unlike other studies (Chen et al., 2023; Dong et al., 2024), We found that the use of aspirin was associated with a lower risk of mortality in SALI patients without coexisting coronary artery disease (CAD), hypertension, or diabetes. We believe that this difference may be attributed to the relatively small sample size of our study compared to other studies and the small and uneven sample sizes of each subgroup after the subgroup analysis, which contributed to significant heterogeneity in the results. Additionally, we found a significant positive linear correlation between INR and the prognosis of SALI patients. Meantime, Aspirin did not demonstrate significant efficacy in SALI patients with an INR greater than 2.1. This indicates that when SALI patients exhibit severe coagulopathy, the imbalance in the “inflammatory-coagulation cascade” may exceed the regulatory capacity of aspirin. Therefore, careful assessment based on individual differences is essential to ensure the safety and effectiveness of aspirin use.

Our study has several limitations. First, this study was retrospective, and although potential confounding variables were balanced or adjusted using PSM or multivariate regression, unmeasured confounding factors may still have influenced the results. Residual confounding has the potential to introduce bias, leading to an overestimation of the effect size. Therefore, the findings should be interpreted with caution. Second, there were individual differences in patients using aspirin and this difference was ignored in this study. Third, the sample size of the study was small, while our subgroup analysis showed a better benefit of aspirin in certain specific SALI patients, this part of the results requires further investigation considering the small sample size of each subgroup variable and no adjustment for confounding factors. Fourth, a causal relationship between aspirin used and outcome in patients with SALI could not be established. Fifth, there is no gold standard for the diagnosis of SALI, and it is often caused by multiple factors such as ischemia, hypoxia, and biliary obstruction. Therefore, the SALI patients included in this study may represent only a subtype of SALI, and further research is needed to determine whether the findings are fully applicable to all SALI patients. Sixth, although this study imputed missing values through multiple imputation and the missForest method, and selected the dataset that was closest to the distribution of the original data to reduce missing bias, it cannot fully address the risk of violating the missing -at-random (MAR) assumption. Seventh, the MIMIC-IV data lack text records of the reasons for aspirin use. And the absence of data on indications and treatment courses may introduce indication bias. Prospective studies are needed for verification in the future. Eighth, the data of this study are from a tertiary medical center in the United States. Although the MIMIC-IV database already includes multiple ethnic groups, there is still a possibility that the generalizability of the conclusions may be limited. In the future, the generalization of the conclusions requires verification in other regions. Nineth, while we employed advanced causal inference methods to address time-dependent confounding, the observational nature of our data fundamentally limits causal interpretation of treatment timing effects. Small sample sizes in critical subgroups likely introduced residual bias, and immortal time bias remains an insurmountable challenge without prospective treatment assignment. Future randomized trials stratifying by therapeutic time windows are urgently needed to clarify aspirin’s temporal effects in SALI. Therefore, the results of this study need to be further verified through large - scale, prospective, multicenter studies.

## 5 Conclusion

The use of aspirin is an independent protective factor for the 30-day mortality in SALI patients, and its use can reduce the 30-day mortality in these patients, regardless of aspirin dose and timing. However, considering the limitations of this study, the clinical application of aspirin should be carefully selected based on individual differences, including indicators such as INR.

## Data Availability

The original contributions presented in the study are included in the article/Supplementary Material, further inquiries can be directed to the corresponding author.

## Abbreviations

SALI: Sepsis-associated liver injury; MBP: Mean Blood Pressure; SpO_2_: Oxygen Saturation; WBC: White Blood Cell; INR: International Normalized Ratio; ALT: Alanine Aminotransferase; ALP: Alkaline Phosphatase; AST: Aspartate Aminotransferase; LDH: Lactate Dehydrogenase; PaO_2_: Partial Pressure of Arterial Oxygen; PaCO_2_: Partial Pressure of Arterial Carbon Dioxide; FiO_2_: Fraction of Inspired Oxygen; CCI: Charlson Comorbidity Index; SOFA: Sequential Organ Failure Assessment; SAPS II: Simplified Acute Physiology Score II; CAD: Coronary Artery Disease; AKI: Acute Kidney Injury; RRT: Renal Replacement Therapy; PSM: Propensity Score Matching; CI, confidence interval; HR, Hazard Ratio.
