# Supplementary material for: Aspirin is associated with improved 30-day mortality in patients with sepsis-associated liver injury: a retrospective cohort study based on MIMIC IV database
Source: Front Pharmacol. 2025 Mar 4;16:1514392. doi: 10.3389/fphar.2025.1514392 (PMC11913821; doi:10.3389/fphar.2025.1514392)
Supplement: Supplementary file 1 [file DataSheet1.docx]

Aspirin is Associated with Improved 30-day Mortality in Patients with Sepsis-associated Liver Injury: A retrospective Cohort Study Based on MIMIC IV Database

Jianbao Wang^1,2,†^, Xuemei Hu^1,2,†^, Susu Cao^1,2,†^, Yiwen Zhao^2,3^, Mengting Chen^1,2^, Tianfeng Hua^1,2^, Min Yang^1,2,^*

1 The Second Department of Critical Care Medicine, The Second Affiliated Hospital of Anhui Medical University, 678 Furong Road, 230601, Hefei, Anhui Province, China

2 Laboratory of Cardiopulmonary Resuscitation and Critical Care, The Second Affiliated Hospital of Anhui Medical University, 678 Furong Road, 230601, Hefei, Anhui Province, China

3 Department of Pediatrics, The Second Affiliated Hospital of Anhui Medical University, 678 Furong Road, 230601, Hefei, Anhui Province, China

^†^Jianbao Wang, Xuemei Hu, and Susu Cao contributed equally to the study.

***Correspondence:** Min Yang, [yangmin@ahmu.edu.cn](mailto:yangmin@ahmu.edu.cn)

Supplementary Material

Catalogue

Supplementary Figure S1: Data Preparation 2

Supplementary Figure S2: Kaplan-Meier Survival Analysis by Aspirin before PSM 3

Supplementary Figure S3: Kaplan-Meier Survival Analysis by Dosage of Aspirin 4

Supplementary Figure S4: Kaplan-Meier Survival Analysis by Timing of Aspirin 5

Supplementary Figure S5: Subgroup Analysis of the Patients with SALI 6

Supplementary Figure S6: Unusual Rates for Different Variables 7

Supplementary Figure S7: Absolute Platelet Change and Incidence of DIC in Two Groups 7

Supplementary Table S1: Categorized Variables and Direction of Values 8

Supplementary Table S2: Association between the Timing of Aspirin Use and Mortality 9

Supplementary Table S3: Absolute Platelet Changes within 24 Hours in Two Groups 9


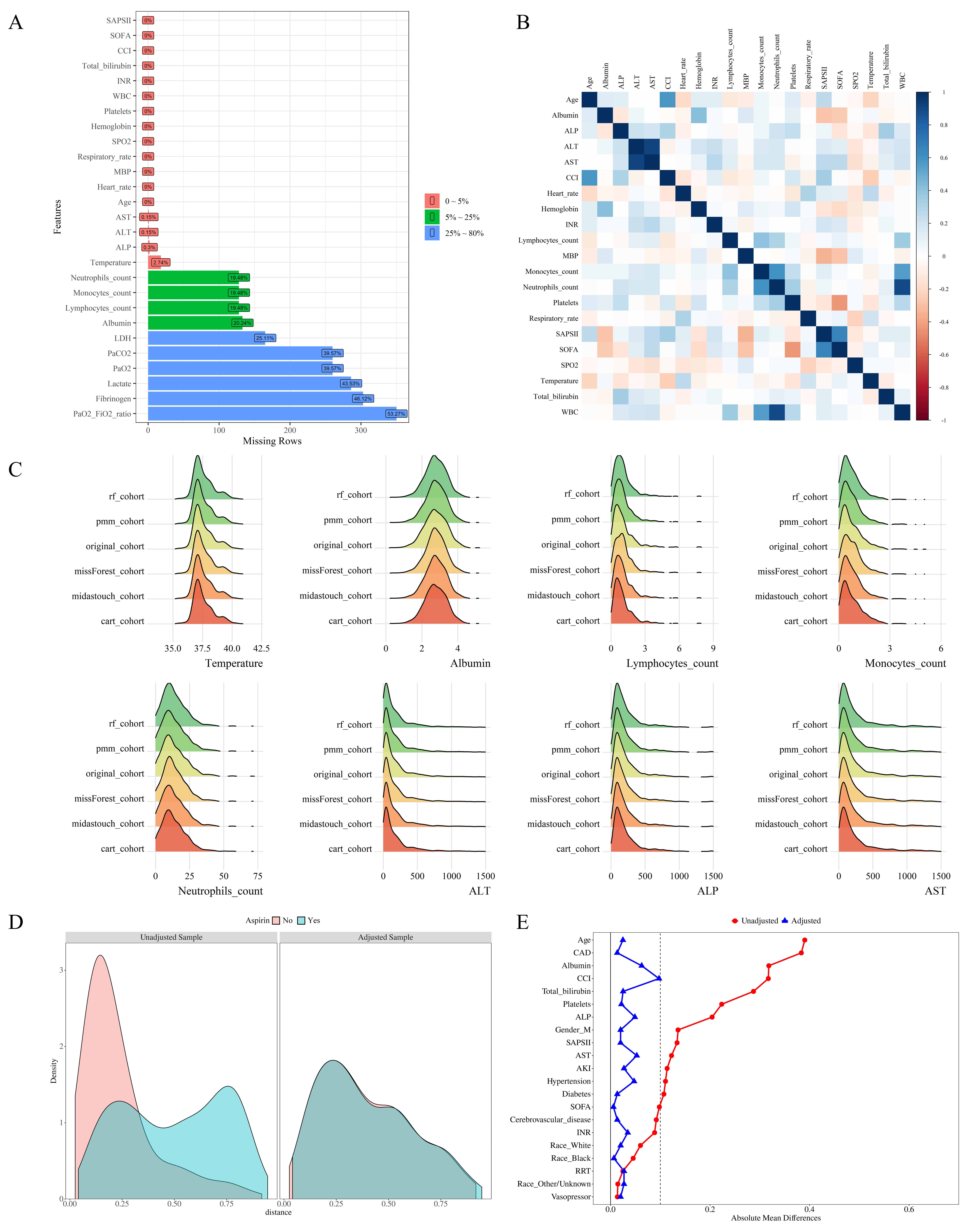


**Supplementary Figure S1: Data Preparation.** Figure (A) provides the proportion of missing values for each variable, variables with more than 25% missing data were removed. Figure (B) shows the heatmap of the correlation between clinical features. The heatmap shows a deeper color (blue or red) when the Spearman rank order correlation coefficient is greater in a positive or negative direction. A high correlated pair of variables is defined as the Spearman coefficient > 0.7. Figure (C) shows the distribution curves of the imputed data for each imputation method, and the imputed cohort that was most similar to the original cohort was selected for data analysis. Figures (D) and (E) show the data distribution before and after PSM. Confounding variable balance was assessed using absolute mean differences and meaningful imbalances were set at values higher than 10%. SALI: Sepsis-associated liver injury; MBP: Mean Blood Pressure; SpO_2_: Oxygen Saturation; WBC: White Blood Cell; INR: International Normalized Ratio; ALT: Alanine Aminotransferase; ALP: Alkaline Phosphatase; AST: Aspartate Aminotransferase; LDH: Lactate Dehydrogenase; PaO_2_: Partial Pressure of Arterial Oxygen; PaCO_2_: Partial Pressure of Arterial Carbon Dioxide; FiO_2_: Fraction of Inspired Oxygen; CCI: Charlson Comorbidity Index; SOFA: Sequential Organ Failure Assessment; SAPS II: Simplified Acute Physiology Score II; CAD: Coronary Artery Disease; AKI: Acute Kidney Injury; RRT: Renal Replacement Therapy; PSM: Propensity Score Matching.


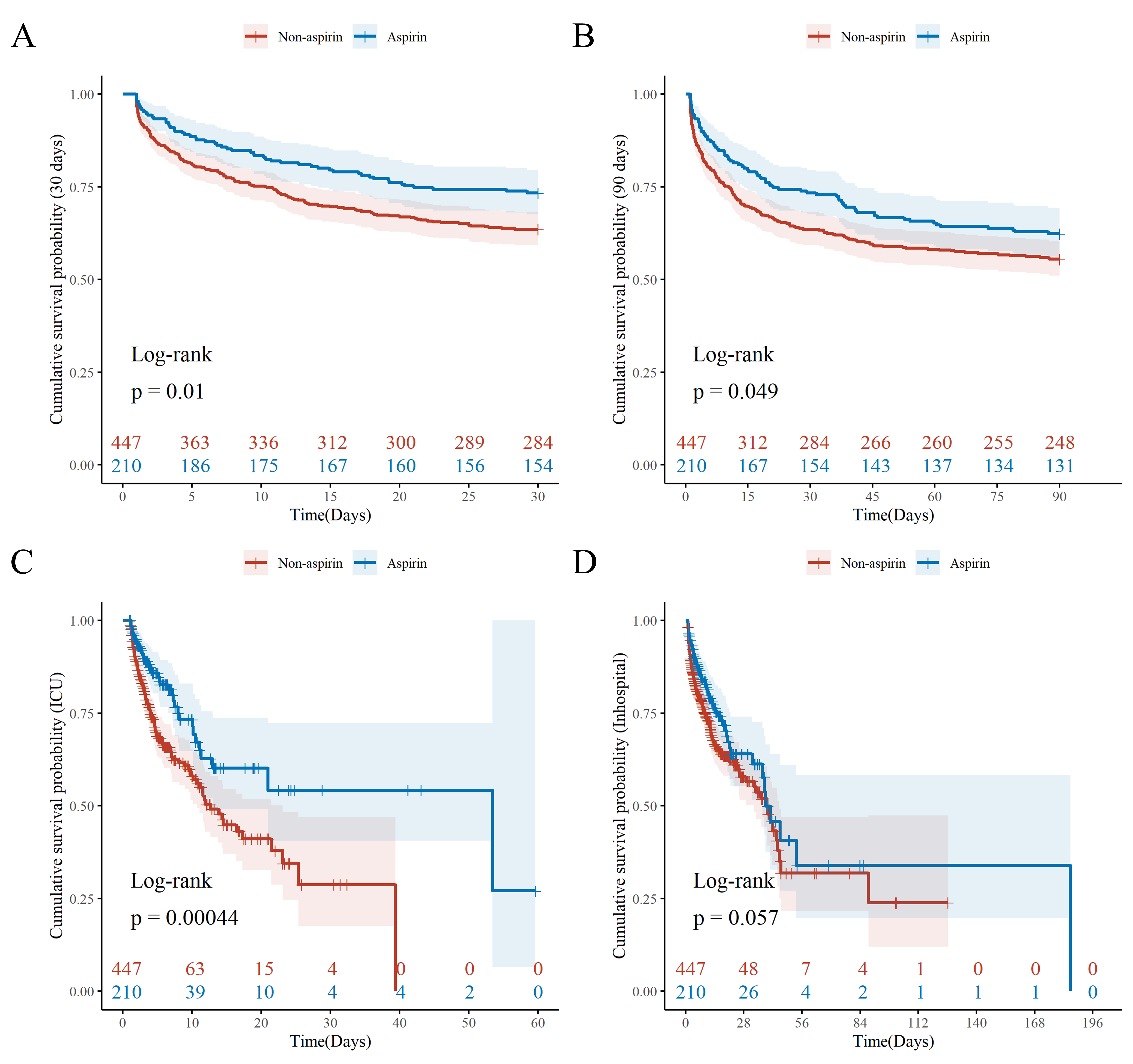


**Supplementary Figure S2: Kaplan-Meier Survival Analysis by Aspirin before PSM.** Kaplan–Meier curves (log-rank test) are plotted for 30-day mortality (A), 90-day mortality (B), ICU mortality (C), and in-hospital mortality (D), grouped by aspirin use. The X-axis denotes the time (days) in ICU and the Y-axis denotes the cumulative survival probability. ICU: Intensive Care Unit; PSM: Propensity Score Matching. *P* value < 0.05 is considered statistical significance.


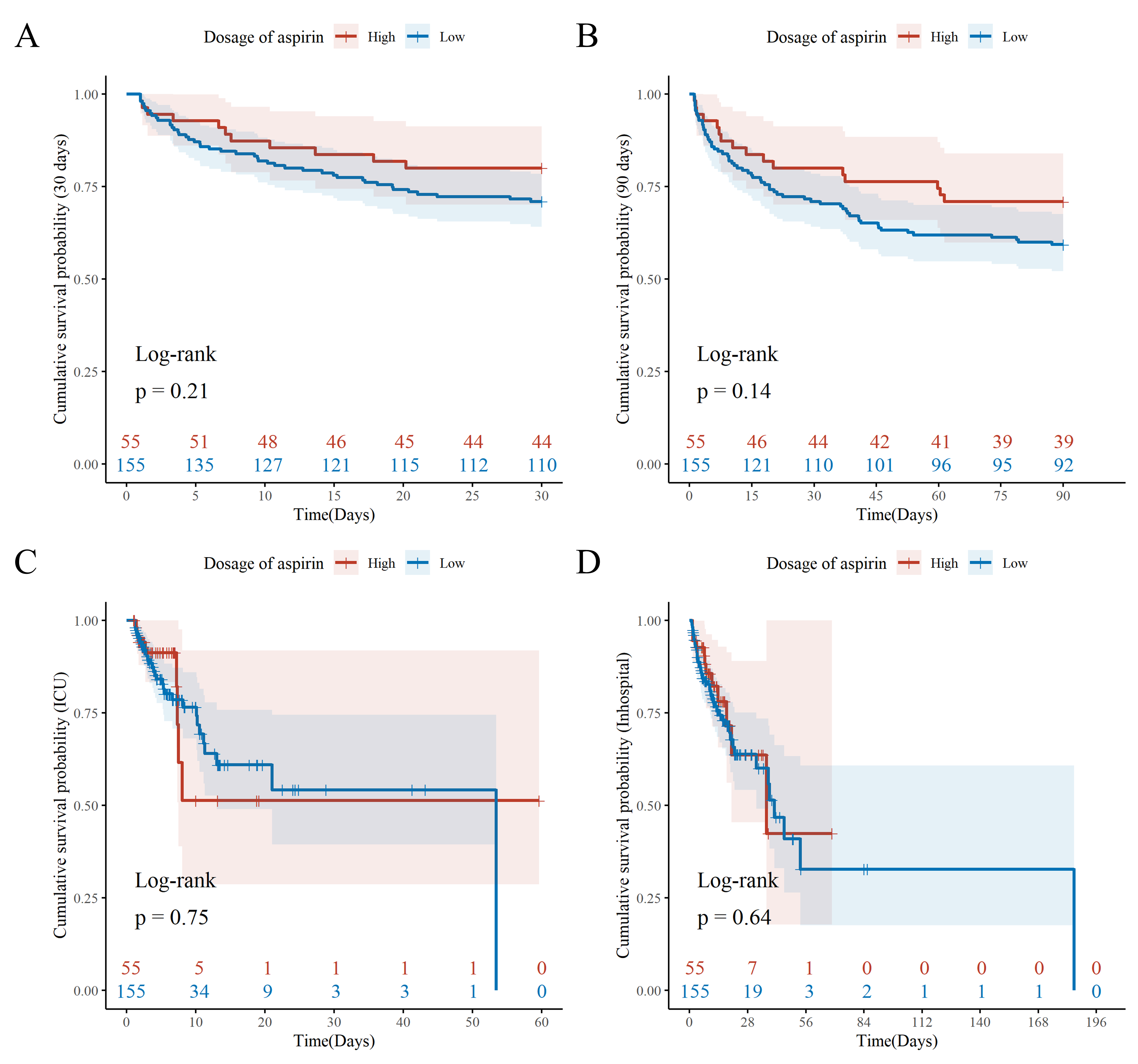


**Supplementary Figure S3: Kaplan-Meier Survival Analysis by Dosage of Aspirin.** Kaplan–Meier curves (log-rank test) are plotted for 30-day mortality (A), 90-day mortality (B), ICU mortality (C), and in-hospital mortality (D), grouped by aspirin use. The X-axis denotes the time (days) in ICU and the Y-axis denotes the cumulative survival probability. ICU: Intensive Care Unit. *P* value < 0.05 is considered statistical significance.


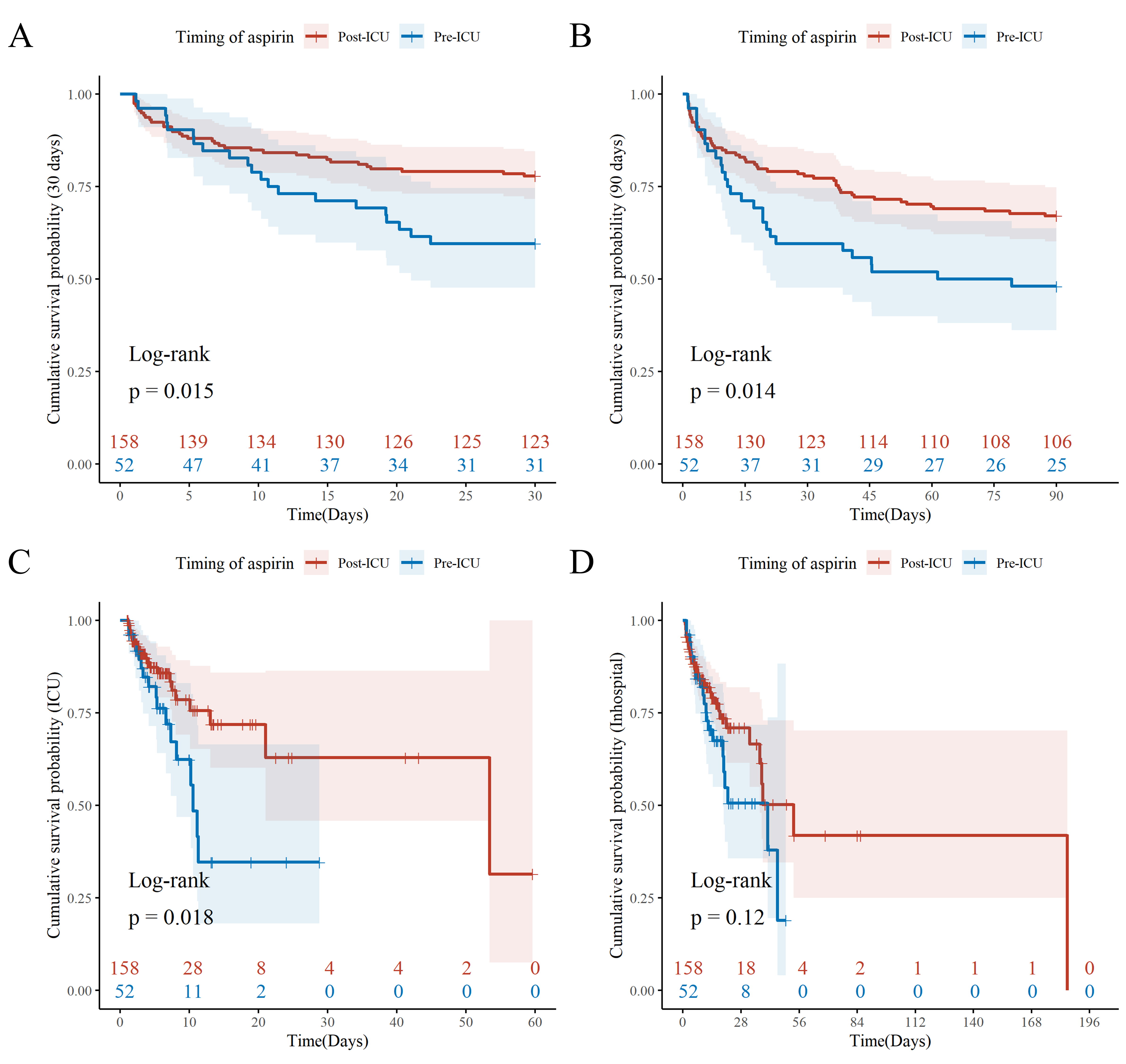


**Supplementary Figure S4: Kaplan-Meier Survival Analysis by Timing of Aspirin.** Kaplan–Meier curves (log-rank test) are plotted for 30-day mortality (A), 90-day mortality (B), ICU mortality (C), and in-hospital mortality (D), grouped by aspirin use. The X-axis denotes the time (days) in ICU and the Y-axis denotes the cumulative survival probability. ICU: Intensive Care Unit. *P* value < 0.05 is considered statistical significance.


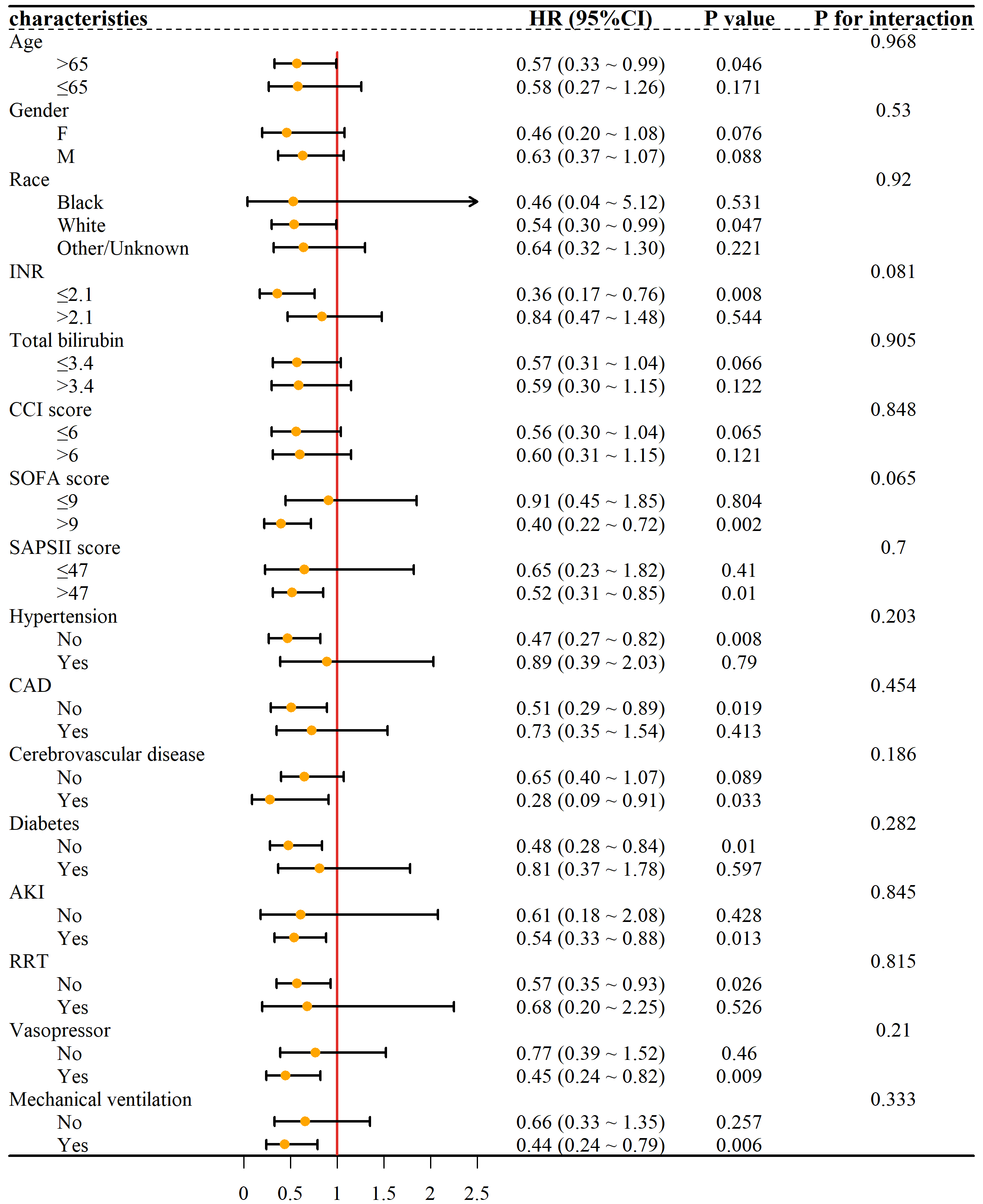


**Supplementary Figure S5: Subgroup Analysis of the Patients with SALI.** We used a stratified Cox proportional hazards model for subgroup analysis, with the non-aspirin group as the common control. For continuous variables, all were divided based on the median, except for age, which was categorized into two subgroups at 65 years. INR, total bilirubin, CCI score, SOFA score, and SAPS II score were divided into two groups according to medians. SALI: Sepsis-associated liver injury; SOFA: Sequential Organ Failure Assessment; SAPA II: Simplified Acute Physiology Score II; INR: International Normalized Ratio; CCI: Charlson Comorbidity Index; CAD: Coronary Artery Disease; AKI: Acute Kidney Injury; RRT: Renal Replacement Therapy; HR: hazard ratio; CI: confidence interval. *P* value < 0.05 is considered statistical significance.


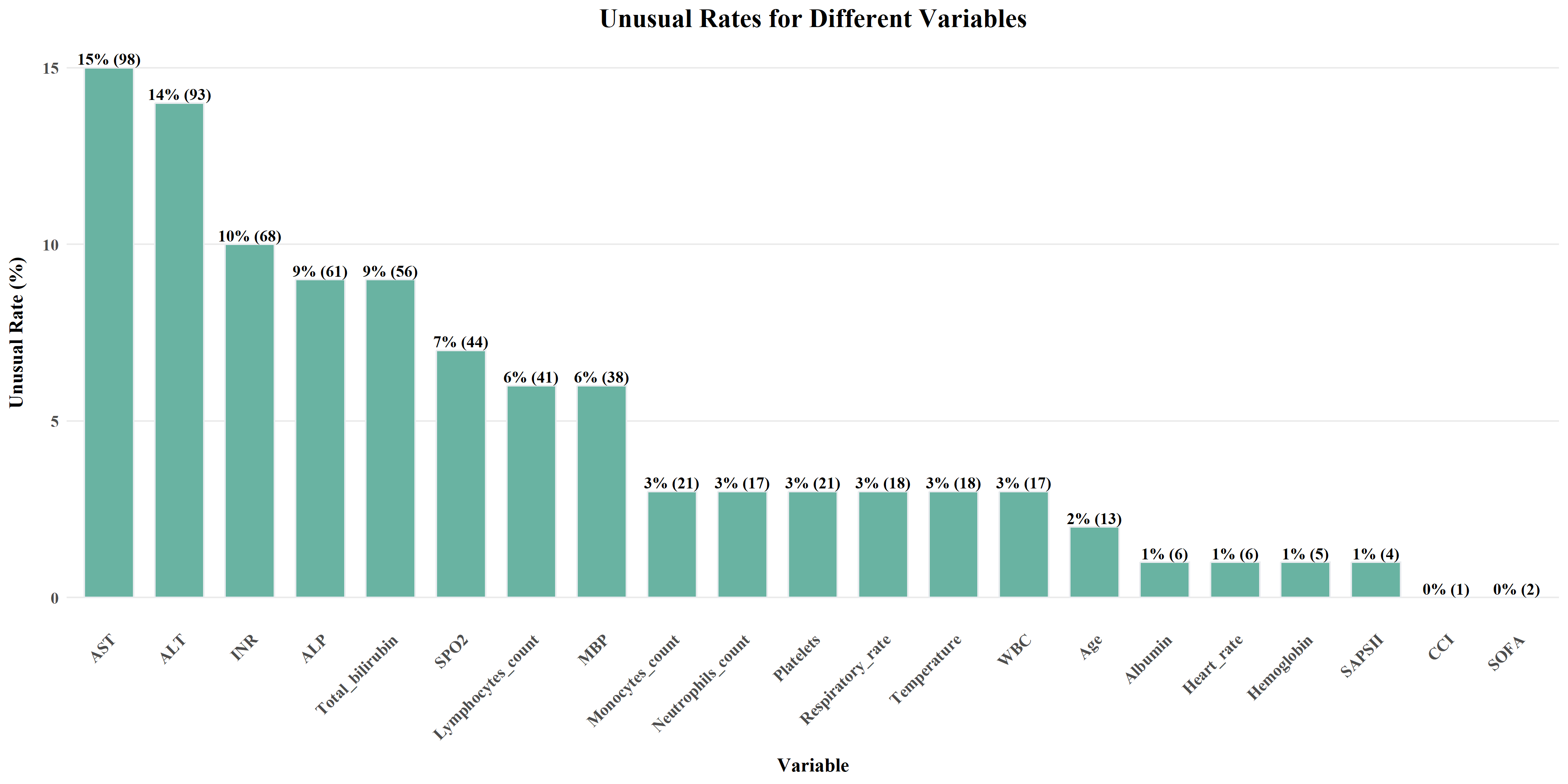


**Supplementary Figure S6: Unusual Rates for Different Variables.** MBP: Mean Blood Pressure; SpO_2_: Oxygen Saturation; WBC: White Blood Cell; INR: International Normalized Ratio; ALT: Alanine Aminotransferase; ALP: Alkaline Phosphatase; AST: Aspartate Aminotransferase; CCI: Charlson Comorbidity Index; SOFA: Sequential Organ Failure Assessment; SAPS II: Simplified Acute Physiology Score II.


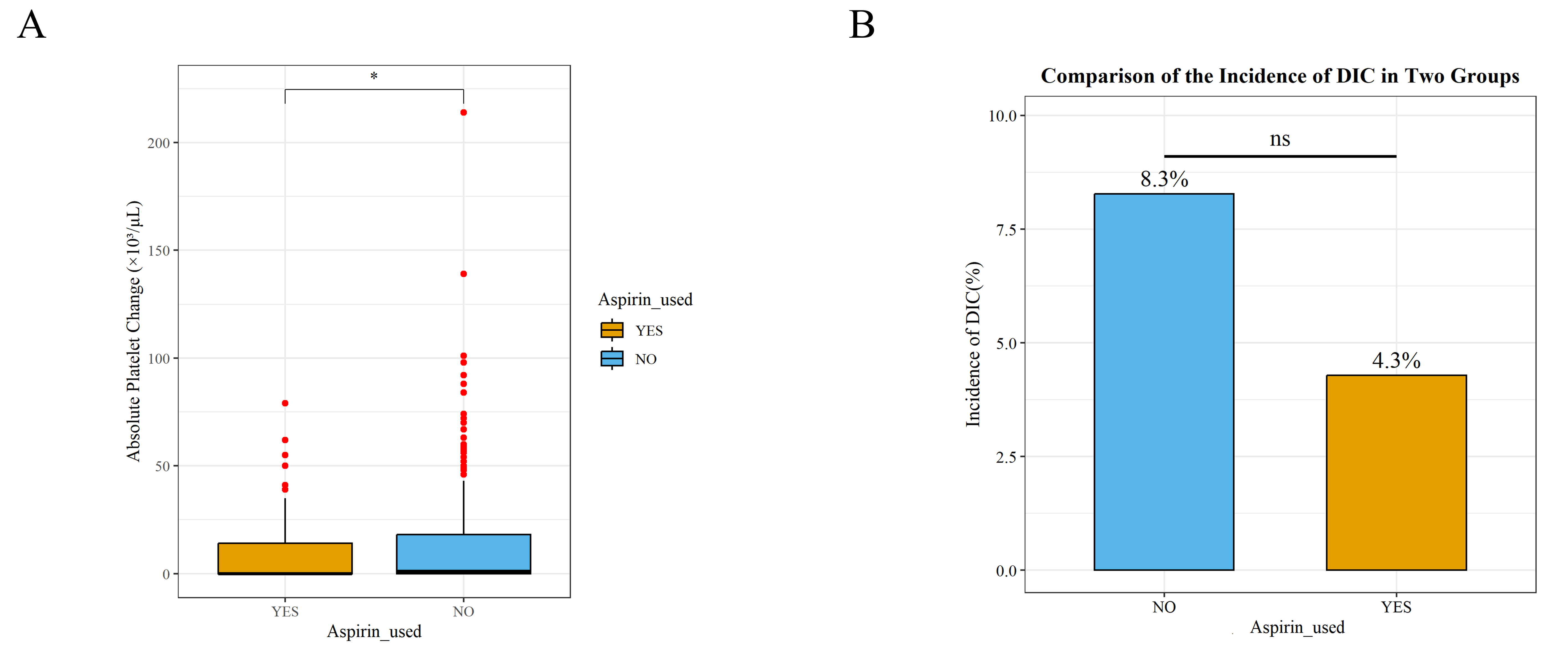


**Supplementary Figure S7: Absolute Platelet Change and Incidence of DIC in Two Groups.** *: *P* < 0.05; ns: non-significant; DIC: Disseminated Intravascular Coagulation.

| **Supplementary Table S1: Categorized Variables and Direction of Values** | | | | |
| --- | --- | --- | --- | --- |
| Categories | Variables | Unit of measurement | Direction of abnormality | Direction of extraction |
| Inflammatory | Temperature | ℃ | Higher | Max |
| Inflammatory | WBC | 10^3/uL | Higher | Max |
| Inflammatory | Monocytes count | 10^3/uL | Higher | Max |
| Inflammatory | Lymphocytes count | 10^3/uL | Higher | Max |
| Inflammatory | Neutrophils count | 10^3/uL | Higher | Max |
| Cardiovascular | Heart rate | bpm | Higher | Max |
| Cardiovascular | Mean blood pressure | mmHg | Lower | Min |
| Cardiovascular | Lactate | Mmol/L | Higher | Max |
| Respiratory | PaO_2_ | mmHg | Lower | Min |
| Respiratory | PaCO_2_ | mmHg | Higher | Max |
| Respiratory | PaO_2_FiO_2_ratio | mmHg | Lower | Min |
| Respiratory | Respiratory rate | bpm | Higher | Max |
| Respiratory | SPO_2_ | % | Lower | Min |
| Hematologic | Hemoglobin | g/dL | Lower | Min |
| Coagulatory | Platelets | 10^3/uL | Lower | Min |
| Coagulatory | Fibrinogen | mg/dL | Lower | Min |
| Coagulatory | INR | / | Higher | Max |
| Liver | Total bilirubin | mg/dL | Higher | Max |
| Liver | ALT | U/L | Higher | Max |
| Liver | ALP | U/L | Higher | Max |
| Liver | AST | U/L | Higher | Max |
| Liver | LDH | U/L | Higher | Max |
| Liver | Albumin | g/dL | Lower | Min |
| Other | Age | year | Higher | Max |
| Comorbidity score | CCI | score | Higher | Max |
| Severity score | SOFA | score | Higher | Max |
| Severity score | SAPS II | score | Higher | Max |

MBP: Mean Blood Pressure; SpO_2_: Oxygen Saturation; WBC: White Blood Cell; SOFA: Sequential Organ Failure Assessment; SAPS II: Simplified Acute Physiology Score II; INR: International Normalized Ratio; ALT: Alanine Aminotransferase; ALP: Alkaline Phosphatase; AST: Aspartate Aminotransferase; LDH: Lactate Dehydrogenase; PaO_2_: Partial Pressure of Arterial Oxygen; PaCO_2_: Partial Pressure of Arterial Carbon Dioxide; FiO_2_: Fraction of Inspired Oxygen; CCI: Charlson Comorbidity Index; CAD: Coronary Artery Disease; AKI: Acute Kidney Injury; RRT: Renal Replacement Therapy; PSM: Propensity Score Matching.

| **Supplementary Table S2: Association Between the Timing of Aspirin Use and Mortality** | | | |
| --- | --- | --- | --- |
| Mortality | Model | HR (95 % CI) | P value |
| 30-day mortality | Unadjusted | 1.93 (1.12 ~ 3.32) | 0.017 |
|  | Multivariate adjusted | 1.87 (0.98 ~ 3.55) | 0.056 |
| 90-day mortality | Unadjusted | 1.78 (1.12 ~ 2.84) | 0.015 |
|  | Multivariate adjusted | 1.54 (0.90 ~ 2.64) | 0.115 |
| ICU mortality | Unadjusted | 2.11 (1.12 ~ 3.98) | 0.021 |
|  | Multivariate adjusted | 2.62 (1.17 ~ 5.88) | 0.019 |
| In-hospital mortality | Unadjusted | 1.54 (0.89 ~ 2.64) | 0.120 |
|  | Multivariate adjusted | 1.46 (0.76 ~ 2.79) | 0.253 |

Multivariate adjusted: Adjusted for age, gender, race, platelets, albumin, INR, total bilirubin, ALP, AST, CCI score, SOFA score, SAPS II score, hypertension, CAD, cerebrovascular, diabetes, AKI, RRT, vasopressor, and mechanical ventilation. SALI: Sepsis-associated liver injury; SOFA: Sequential Organ Failure Assessment; SAPS II: Simplified Acute Physiology Score II; INR: International Normalized Ratio; ALP: Alkaline Phosphatase; AST: Aspartate Aminotransferase; CCI: Charlson Comorbidity Index; CAD: Coronary Artery Disease; AKI: Acute Kidney Injury; RRT: Renal Replacement Therapy; PSM: Propensity Score Matching; ICU: Intensive Care Unit; HR: Hazard Ratio; CI: Confidence Interval. *P* value < 0.05 is considered statistical significance.

| **Supplementary Table S3: Absolute Platelet Changes within 24 Hours in Two Groups** | | | | |
| --- | --- | --- | --- | --- |
| Aspirin used | Time (Hours) | Absolute Platelet Change (×10³/μL) | *t* | *P* value |
| YES | 24 | 8.82±14.64 | -2.067 | 0.04 |
| NO | 24 | 12.36±21.94 |  |  |
